# Exploring the transformative potential of design thinking pedagogy in hybrid setting: a case study of field exercise course, Japan

**DOI:** 10.1007/s12564-022-09776-3

**Published:** 2022-08-29

**Authors:** Sadaf Taimur, Motoharu Onuki, Huma Mursaleen

**Affiliations:** Graduate Program in Sustainability Science – Global Leadership Initiative (GPSS-GLI), University of Tokyo, Kashiwa, Japan

**Keywords:** Transformative learning, Higher sustainability education, Design thinking, Pedagogy

## Abstract

The current research explores the transformative potential of Design Thinking (DT) pedagogy in Higher Sustainability Education (HSE) in a hybrid (mix of online and face-to-face) setting. The case study examined a Field Exercise in Sustainability Science (FESS) course, using DT pedagogy, in a HSE program at a university in Japan. The critical transformative learning experiences (i.e., signs of transformative learning) were captured to investigate whether DT pedagogy has allowed learners to challenge their perspectives and go through the transformative learning experience. The qualitative case study captures the critical transformative learning experiences by collecting students’ perspectives through semi-structured interviews, reflections, and class observations. As a result, the research successfully captured the signs of transformative learning, where each student went through multidimensional and embodied transformative experiences. Furthermore, the results show how DT as a pedagogy can support transformative learning in HSE by encouraging disorienting dilemmas, promoting reflection and discourse, fostering relationships, providing context, and offering an engaging experience. DT as a pedagogy can bring transformative learning into practice in HSE, and it can be implemented effectively using a digital or hybrid learning approach without a need for complex structural changes. The paper can provide concrete lessons for practice and curriculum development to bring transformative learning into digital teaching practice in HSE.

## Introduction

The unsustainable practices of humankind have led to various challenges, including pollution, illnesses, inequalities, poverty, wars, climate change—all these problems are “wicked.” “Wicked” problems are the complex issues that lack definition and are ill-formulated, where information is confusing, where many stakeholders with conflicting interests are involved in decision making, and where there is confusion regarding the consequences of these challenges on the whole system (Buchanan, 1992; Earle & Leyva-de, 2021; Rittel & Webber, 1973). Sustainability as an emerging field has been developed to deal with these “wicked” sustainability challenges via transformational action (Blackstock & Carter, 2007; Grunwald, 2004; Talwar et al., 2011). Furthermore, education has been linked to change and the need to achieve sustainability globally since the 1970s (Sterling, 2004). Therefore, Sustainability Education (SE) can equip learners to deal with sustainability challenges. UNESCO (2017) defined SE, also known as Education for Sustainable Development (ESD), as education that intends to empower students to make informed decisions and responsible actions to ensure economic feasibility, just society, and environmental integrity while respecting cultural diversity—for the present and future generations.

### Higher sustainability education & transformative learning

SE is needed at all levels of education, but those who have access to higher education should be well-versed with sustainability to deal with complex sustainability challenges in local and global communities (Burns, 2009). Higher education institutions play a pivotal role in facilitating transitions toward sustainable societies and the environment (Orr, 2004; Sachs et al., 2019; Weiss et al., 2021). SE in the higher education setting, termed as Higher Sustainability Education (HSE), aims to educate future leaders who can build a sustainable society (Onuki & Mino, 2009). Encouraging the transformation of higher education can help contribute to reorienting societies toward sustainability or sustainable development. Universities have enormous potential to lead in questioning the status quo, challenging paradigms, and practicing new ways of learning, teaching, thinking, and living.

The interdisciplinary field of sustainability in the higher education setting demands change as moving toward sustainability is impossible with the current (transmissive^1^) approaches (Moore, 2005). The transmissive approaches do not equip learners to deal with issues so complex and deeply challenging as is the case with sustainability (Sterling, 2010). Therefore, it is crucial to shift the ways of thinking and learning—to be more connective, systemic, holistic, and ecological (Sterling, 2001). This shift requires moving away from the teacher-centered transmissive approach to the learner-centered transformative approach to enable learning, which can explore the depth of things and bring about a paradigmatic shift from transmissive to transformative learning (Burns, 2011; Cress, 2003).

Mezirow regarded transformative learning as the kind of learning that nurtures autonomous thinking as in contemporary societies (Mezirow, 1997). Transformative learning is defined as

[. . .] learning that transforms problematic frames of reference – sets of fixed assumptions and expectations (habits of mind, meaning perspectives, mindsets) – to make them more inclusive, discriminating, open, reflective, and emotionally able to change (Mezirow, 2003, p. 58).

Transformative learning is about awareness of one's own and others' perspectives and expectations, subsequently evaluating their relevance (critically) for interpretation (Mezirow, 2000). Transformative learning is about understanding learning as a process of using a prior interpretation to construe a new or revised interpretation of the meaning of one's experience to guide future action (Mezirow, 1996). Adults view their life with limited perspectives based on their limited experiences that shape their beliefs (truth). When these experiences are expanded through transformative learning, individuals may challenge their existing beliefs and gain new perspectives (also recognized as transformation) (Nerstrom, 2014).

Various conceptualizations of transformative learning have been proposed encompassing individual and social purposes, e.g., empowerment, autonomy, individualization, social action, ecological consciousness, democracy, and citizenship (Cranton & King, 2003; Mezirow, 1997, 2003). The general aims and broad understanding of transformative learning are to contribute to social transformation (change) through education, making it appealing to SE (Aboytes & Barth, 2020). In HSE, transformative learning transforms learners' values and perspectives to embrace sustainability as a new paradigm or a lens through which to view the world and make a change (Burns, 2009, 2015). Students question their paradigm when exposed to new (expanded) experiences and reconstruct it by shifting their values and perspectives. Transformative learning can develop the capacities and qualities of individuals, groups, and communities to meet the challenges linked to sustainability (Wals, 2011), and it can enhance the effectiveness of SE in the higher education setting (Taimur & Onuki, 2020).

### Implementing transformative learning via pedagogy in higher sustainability education

Different aspects of transformative learning have been discussed in the literature, but they share an emphasis on the learning environment and processes (pedagogy^2^), focusing on the discourse, critical reflection, and experience (Mezirow, 2003; Taimur & Onuki, 2020; Taylor, 2007). Therefore, it is critical to focus on pedagogy to bring transformative learning into HSE practice (Taimur, 2020; Taimur & Onuki, 2020, 2022) and move away from transmissive pedagogy to transformative pedagogy. Transformative pedagogy is defined as a pedagogy that can construct learning environments and processes that expose learners to transformative learning experiences (Taimur & Onuki, 2022); this enables students to probe their assumptions critically, grapple with social challenges, and engage in social action (Meyers, 2008). This kind of pedagogy is usually organized around problem-oriented approaches and action-oriented projects (Kitano, 1997; Meyers, 2008; Nielsen, 2020); hence, problem-based learning can offer such experiences. Some studies showcased problem-based learning as transformative pedagogy (e.g., Cavanagh, 2019; Nielsen, 2020; Wynn & Okie, 2017) and highlighted practices for implementing problem-oriented, project-based learning in HSE (e.g., Birdman et al., 2021; Caniglia et al., 2018). However, the potential of problem-based learning is yet not realized in HSE due to the limited emphasis on approaches promoting such learning (Leal Filho et al., 2018).

### Design thinking as pedagogy in higher sustainability education

Design thinking (DT) is defined as a human-centered problem-solving approach to deal with “wicked” challenges (Buchanan, 1992; Dam & Siang, 2018; von Thienen et al., 2014), which can be utilized as a pedagogy in an educational context (Luka, 2014). DT pedagogy is beneficial because it allows students to work in multidisciplinary teams, train them to deal with complex challenges, and enact design-led and positive change in the world (Munyai, 2016; Rauth et al., 2010). DT pedagogy can implement problem-based learning in higher education (Acharya et al., 2021). However, it is different from other problem-based learning approaches as it allows participants to frame the problem instead of providing them with pre-determined problems, and the iterative stages of DT pedagogy are supported by explicit strategies (tools) (Melles, 2015). Resolving “wicked” sustainability challenges requires understanding the complexity of the systems (Remington-Doucette 2013), along with continuous, reflexive, and adaptive responses over time (McCrory et al., 2021). Therefore, problem framing becomes crucial to establish a shared understanding to deal with the sustainability challenges. DT offers to instill traits that are beneficial for solving pressing sustainability challenges and making a difference, i.e., creative problem-solving, collaborating across disciplines, optimism, and experimenting with solutions (to learn and adapt quickly) (Shapira et al., 2015). Correspondingly, the human-centered nature of DT is socially situated in values and sense-making; a design thinker being a transformative learner, sees beyond others, can draw from diverse perspectives, and imagine innovative solutions that are better than existing choices (Avsec & Ferk Savec, 2021). DT can promote transformative learning, as it (a) supports focus on values; (b) is multidimensional and multidisciplinary; (c) produces clear resolves for multiple stakeholders and produces learning outcomes that can address these resolves; (d) creates space for enabling interdisciplinary reflective discourse, (e) promotes social critique and stakeholders engagement to strengthen the relationship between learning experiences, curriculum, needs of students and other stakeholders, and (f) encourages risk-taking and exploration (Avsec & Ferk Savec, 2021; Benson & Dresdow, 2014). Hence, DT can be used as a (problem-based) transformative pedagogy to promote transformative learning in HSE and train sustainability leaders effectively. When DT is applied as a pedagogy in HSE, it aims to allow learners (in diverse^3^ teams) to identify a sustainability challenge in a particular area/region/community and then develop an innovative solution to deal with the identified challenge.

**Fig. 1:**
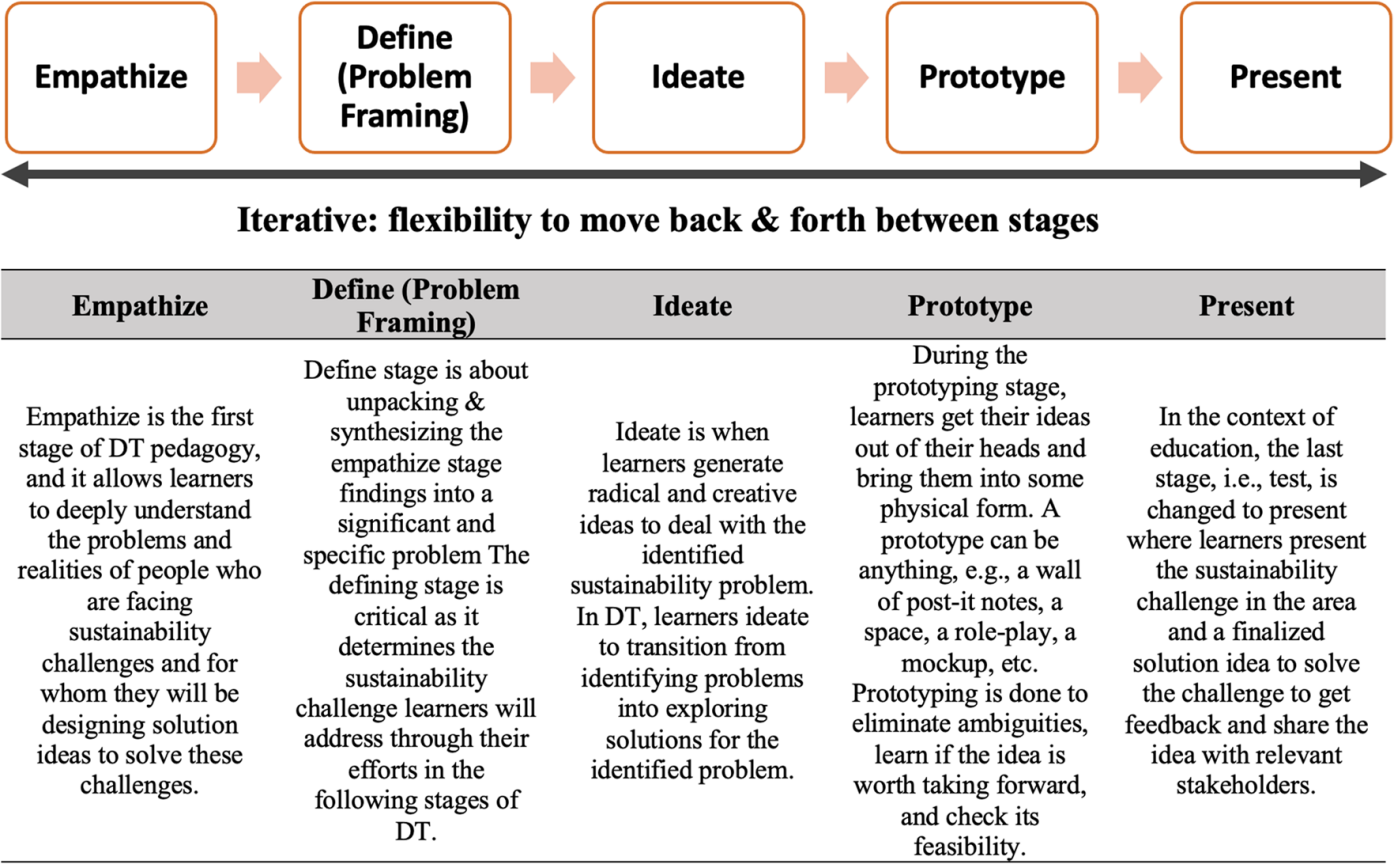
Five stages of design thinking pedagogy (Adapted from Plattner, 2,010)

Several models have been created to demonstrate DT based on Simon’s (1969) DT process but the most notable among them, in the context of education (Melles, 2015), is the one developed by the Institute of Design at Stanford D-School (Plattner, 2010; Plattner et al., 2009). This iterative DT process emerges from human-centered design principles and has five stages, i.e., empathize, define, ideate, prototype, and test. We considered five-staged DT pedagogy in the current research, based on Plattner (2010). The last stage, i.e., the test in the original model was converted to present—as DT was being used as a pedagogy in higher education settings, which entails communicating what is designed to enhance understanding and engaging multiple stakeholders (Benson & Dresdow, 2014). Figure 1 explains the stages of DT pedagogy and highlights that DT is an iterative pedagogy where learners can move back and forth between all the stages of DT.

DT has been examined in the context of sustainability and HSE, for example, (a) as a method for sustainability scientists to deal with sustainability challenges (Fischer, 2015); (b) as an approach to tackle sustainability challenges and foster sustainability-oriented innovation development (Buhl et al., 2019); and (c) as an approach to enhance students’ skills to understand, formulate, and solve sustainability problems (Li et al., 2018; Oxenswärdh & Persson-Fischier, 2020). However, more work is needed to explore the transformative potential of DT pedagogy in HSE to understand the transformative learning processes and, subsequently, bring transformative learning into practice.

### Purpose of the study

The purpose of the research is to explore the transformative potential of DT as a pedagogy in HSE course in a hybrid (a mix of digital and face-to-face) setting by capturing the signs of the transformative learning experience. The focus of the current research is not to understand transformative learning as an outcome but to comprehend transformative learning as a process. Focusing on transformative processes can inform learning and curriculum design by improving how adults learn and how they are taught (Snyder, 2008). The current study investigates whether DT pedagogy in the hybrid setting has allowed learners to experience transformative learning by capturing the signs. We used the term “signs” instead of “indicators” to take away the focus from the quantification of experience. These signs are critical transformative learning experiences—these are usually intuitive, interpretive, speculative, and require a dialog (interaction) between those reporting them and those experiencing them (Macintyre et al., 2020). Hence, signs are captured by collecting learners’ perspectives on their learning experiences. When explored as a process, transformative learning has mainly been discussed in terms of the transformative learning phases proposed by Mezirow (Aboytes & Barth, 2020; Nerstrom, 2014; Snyder, 2008). The study uses transformative learning phases (theoretical framework) to identify the signs of transformative learning during the implementation of DT pedagogy in HSE in the hybrid setting. However, it does not restrict the identification of signs to the theoretical model of transformative learning phases (or perspective transformation only).

The study does not intend to identify the universal signs of transformative learning but identifies if DT as a pedagogy sets up the conditions for transformative learning to occur by capturing the signs of transformative learning experiences of the participants in the HSE course. The following research questions (RQs) guided the study:

RQ1: How do participants in a HSE course perceive experiencing transformative learning phases, aligned to the Transformation Helical Model (theoretical framework), while going through the DT pedagogy?

RQ2: How has DT as a transformative pedagogy impacted participants in a HSE course?

RQ2: How has DT pedagogy set up conditions for transformative learning to occur?

The study tries to explore the contextual lessons emerging from the investigation. However, it cannot be said that this investigation has no relevance in other contexts, but those in other contexts can define how important these signs are for their situation. This is also known as case inspired generalization, where the agency of generalization is left to the readers, rather than authors, who can mirror what they read in their context (Macintyre et al., 2020; Wals & Alblas, 1997).

## Design thinking as a pedagogy for transformative learning: theoretical framework

The current research aims to understand transformative learning as a process. Therefore, it employed a Transformation Helical Model (THM) (described below), based on Nerstorm's (2014) phases of transformative learning, to capture transformative learning experiences, while DT is being implemented as a pedagogy in HSE. Nerstrom (2014) developed a model loosely based on Mezirow's (1978) phases of transformative learning after conducting a literature review of over 500 studies and reducing ten phases to four phases. The research adopted Nerstrom's phases of transformative learning to construct the theoretical framework (THM) because it puts a straightforward approach for driving case-study research (Eldaly, 2021), which follows a sequential order and allows researchers to visualize how transformative learning is constructed. Visualizing how transformative learning is constructed simplifies the process and it helps facilitate transformative learning in the real world (Sharpe, 2016) by explaining and strengthening transformative learning experiences (Nerstrom, 2014).

### Transformation helical model

**Fig. 2:**
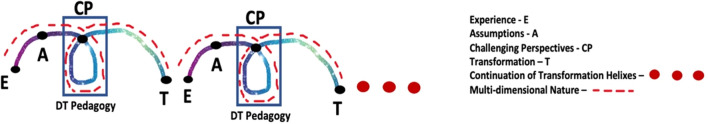
Transformative Helical Model (THM)—Adapted from Nerstrom (2014)

The Transformation Helical Model (THM) intends to demonstrate how transformative learning may occur while DT is being implemented as a pedagogy in HSE. The model presents transformative learning as a helical process of learning phases. These learning phases are comparable to those proposed by Nerstrom (2014) and loosely based on the phases proposed by Mezirow (1978). According to Nerstorm (2014), transformative learning is a continuous cycle of learning, and once it occurs, individuals become more receptive to experiencing that again. The helical process keeps repeating while DT is being implemented as a pedagogy. The model is demonstrated in Fig. 2 (Fig. 2) and explained below.

According to THM, learners come from different backgrounds and have different experiences (E), which stem from the environment and interactions with others throughout their lifetime. Through these experiences (E), learners form assumptions (A) perceive the world around them. When learners are introduced to DT pedagogy in HSE, through DT pedagogical tools, they will question their own assumptions or perspectives (called challenging perspectives (CP)). DT tools in this research are defined as methods with a set of guidelines to support the learning and action of learners during a particular stage of design thinking (adapted from Plattner, 2010). These tools help provide new experiences to the learners, and when these experiences combine with reflection (represented by the loop in the figure), learners challenge their own perspectives (CP) which may lead to adopting new perspectives and acting upon them, also called transformation (T). This transformation (T) becomes their experience (E) or new reality.

THM demonstrates that DT as a pedagogy sets up the learning environment and processes, shown as a blue box (surrounding reflection loop) in Fig. 2, enabling learners to challenge their perspectives which may lead to transformations. THM demonstrates that there may be multiple helixes of transformations throughout the implementation of DT pedagogy, as once transformative learning occurs, individuals are more open to experiencing that again. DT pedagogy encourages transformations by supporting the most crucial phase, i.e., challenging perspectives (CP), of transformative learning. These transformations are unidirectional, as once transformative learning occurs, it is unlikely that individuals will revert to their previous beliefs (Nerstrom, 2014). Consequently, helixes are used to represent the phases of transformation as compared to the circular cycle. The transformation helixes are multidimensional, represented by the dotted line in Fig. 2, where numerous multidimensional (cognitive and affective) transformation helixes are expected to co-occur among learners simultaneously.

THM as a theory-based and evidence-driven model determined the empirical and theoretical rationale of the study, guided the selection of the research methods, and supported the data analysis and interpretation.

## Research approach

The current research used a qualitative case-study research approach. Case study is used to investigate the phenomenon within the context in which it occurs (Creswell, 2009; Merriam, 1988), and contemporary teaching practices in real-life context are effectively captured through it (Yin, 2009). Qualitative case study research helps in providing extensive, rich, and in-depth analysis of the situational context in the real world, covering its associated and distinctive features. The research could be regarded as an "instrumental case-study," as explained by Stake (1995, 2003). The case examined a phenomenon, i.e., the implementation of DT as a pedagogy in HSE classroom in hybrid settings and its corresponding impacts on learners' perceived transformative learning experiences. Stake (1995, 2008) highlighted that the selected case study should facilitate the phenomenon under study and maximize what research can learn. Based on this criterion, a field exercise course from the University of Tokyo in Japan was selected as a case: (a) the course was embedded in the HSE program of the public university in Japan; (b) the course aimed at addressing sustainability challenges in a specific context; (c) the course was open to students from different backgrounds (academic majors and regional background); (d) the course was organized in English; and (e) the course utilized hybrid mode of implementation amid the COVID-19 pandemic due to practical necessities. Thus, it became opportune to re-design the course to implement DT pedagogy and collect data to examine the transformative potential of DT pedagogy.

This study specifically targeted the "Field Exercise in Sustainability Science" (FESS) course at the Graduate Program in Sustainability Science-Global Leadership Initiative (GPSS-GLI) of the University of Tokyo. GPSS-GLI is a HSE academic program offering Masters and Ph.D., which looks forward to dealing with sustainability challenges by developing next generation leaders (Mino et al., 2016). Currently, the program has a FESS course for graduate students that takes Kashiwa no ha as the subject of research interest for participating students. Kashiwa no ha area is located in Kashiwa city, Chiba Prefecture, Japan, and houses one of the campuses of the University of Tokyo. The objective of FESS course is to frame sustainability challenges in Kashiwa no ha area and then propose innovative solution ideas for the framed challenges.

### Course setting

In 2020, to prevent the spread of COVID-19, FESS was implemented using DT as a pedagogy in a hybrid setting—where online educational methods were combined with face-to-face educational methods. The course started on October 14, 2020, and concluded on January 13, 2021. The FESS course engaged the students for 3.5 h every week for 13 weeks in the formal and planned setup.

DT toolkit was created to implement DT as a pedagogy during FESS. The toolkit outlined all 13 sessions of FESS and the tools for each stage of DT to support students' learning and action. Tools for all stages of DT were adapted from IBM (2020), IDEO (2012), Plattner (2010), Tschimmel et al. (2017), and UNLEASH (2019) and tailored to be used for dealing with sustainability challenges and to support discourse and reflection. Discourse is a dialog between at least two people, focusing on content and attempts to justify beliefs by giving and defending reason and by examining the evidence for and against competing viewpoints—it is activated and enhanced by (self and critical) reflection (Aboytes & Barth, 2020; Mezirow, 1994). Discourse and reflection are mandatory conditions for transformative learning to occur (Mezirow, 1991; Taimur & Onuki, 2020); therefore, a critical condition to use DT tools involved working in diverse teams throughout the course, where participants were involved in discourse and reflection. The DT toolkit was prepared in collaboration with the faculty member in charge of the FESS course, with an elaborated explanation of all the sessions and tools to implement DT as a pedagogy in a hybrid/digital setting. The DT tools for guiding the learning and action of learners during each stage of DT were converted into the digital format using Miro boards. Working spaces were designed on the Miro boards for each tool to facilitate virtual teamwork, utilize DT tools, and record the team's progress. A model Miro board prepared to facilitate the course can be seen by following this link: "https://miro.com/app/board/uXjVODwJTtw=/?share_link_id=99009102562". The zoom platform was used to conduct online sessions, and the breakout group function was used to ensure that students worked in their respective teams. The whole setup of the course is seen in Table 1.

**Table 1:** Outline of the FESS course, using DT pedagogy

Stages	Tool	Brief Description of Tool	Mode	Aid/s	Assessment
Design Thinking Pedagogy					
Empathize	Buzz Repository	Creating an undated repository of information to get insights regarding what is happening in the respective area, understanding of changing patterns and currently significant challenges at hand Information can come from published articles, documentaries, reports, newspaper articles, lectures	Online	• Google drive – Team Folder	Peer Feedback -1 Progress Check-1: • Well defined sustainability problem • Problem identification ladder • Empathy Map
	Immersion & Observation	Immersion and observation are about observing the research area and getting immersed in the environment under study	Face-to-face	• Journal • Sticky notes	
	Stakeholder Map	Stakeholder map helps in identifying the shared or opposite interests of the agents involved in or affected by the challenge in a particular area. The objective of stakeholder map is to identify the right stakeholders to engage with in order to understand the problem in depth	Online	• Miro board • Zoom video call	
	Interviews	In order to understand people’s thoughts, motivations, and emotions, it is important to engage with them. Interviews is one of the engagement methods. It helps in collecting Insights from the relevant stakeholders to understand the problem, in depth, from their perspective	Face-to-face & Online	• Journal • Recorder or mobile phone	
Define	Empathy Map	An Empathy Map is one tool to help synthesize observations and draw out unexpected insights from the information collected during the empathize phase. The objective is to analyze the people’s needs and insights “An insight is a friction, contradiction or dilemma that is either a reason why challenge still exists, or a barrier to the adoption of solutions that could mitigate or address that challenge”	Online	• Miro board • Zoom video call	
	Drawing Insights	From all the insights which are collected, after empathize phase, it is important to write an insight to frame a problem. This tool helps in converging to one insight which can be used for problem framing	Online	• Miro board • Zoom video call	
	Problem Identification Ladder	Problem identification ladder is built by asking “Why, How, and Who” questions to dig deep into the broad insight, broad party (people who are affected by the problem), and broad people’s need and produces a well-defined problem framing with a very specific party (people/entity/community/organization facing the problem), need and insight	Online	• Miro board • Zoom video call	
	Problem Identification -Statement	In order to deal with a sustainability challenge from the people’s point of view, it is important to frame the challenge into an actionable problem statement that launches into generative ideation. Problem framing template which is also known as Point of View (PoV) succinctly states your party (community, group, entity, organization affected by the problem), need and insight	Online	• Miro board • Zoom video call	
Ideate	Planting Idea Seeds via Brainstorming	Using this tool ensures that through brainstorming: everyone in the team gets the opportunity to contribute to ideas for solving the identified problem, and all team members build upon each other’s ideas	Online	• Miro board • Zoom video call	Peer Feedback—2 Progress Check-2: • Sketches of 2 shortlisted ideas • Value & Impact vs complexity of 2 shortlisted ideas • Selected idea • Ideation research on selected idea
	Pitching the Idea Concepts	After generating ideas and before down selecting them, each team member should select his/her favorite idea and pitch his/her favorite idea concepts to the rest of the team. This tool helps in pitching innovative ideas and exploring them in a bit more detail	Online	• Miro board • Zoom video call	
	Value and Impact vs Complexity Mapping	This tool helps in assessing the same ideas by how difficult they are to do (implement) vs. their impact and value. This tool is usually used to assess the impact potential of an idea or elements of an idea	Online	• Miro board • Zoom video call	
	Down-Selecting Ideas	This tool provides, multiple, simple ways to help down-select the ideas so that one or a small number of ideas can be focused on to prototype	Online	• Miro board • Zoom video call	
	Ideation Research	Ideation research tool is about researching regional trends, potential competitors, and the successes and failures of similar solutions (if there are any). It helps in learning from the successes and failures of other solutions (already in place) to address the identified sustainability problem	Online	• Miro board • Zoom video call	
Prototype	Party’s Journey Map	This tool helps in drawing the sequential and parallel activities of respective party’s journey with or without solution. The tool pinpoints how solution affects party’s journey and therefore, what is needed to prototype to have the party relate to and test out these changes	Online	• Miro board • Zoom video call	Peer Feedback—3 Progress Check-3: • Party’s journey diagram • Prototype (looks like and works like prototype)
	Rapid Prototyping	Rapid prototyping is a tool for creating physical representation of solution idea cheaply and quickly, which party/ies can experience and react to	Face-to-face	• Flip charts • Markers • Sticky notes • Cardboards • Clay • Blocks/LEGO • Power point or any other software,	
Present	–	–	Online	• Power point • Zoom video call	Final Presentation

FESS course comprised 13 sessions in total. The first two sessions were used for introductions, ice-breaking, and team-building activities. Starting from the third week onward, students used tools to go through various stages of DT pedagogy. By the end of the seventh session, both teams framed a well-defined sustainability problem in Kashiwa no ha area. From the eighth session, teams worked on formulating a solution for the problem they had framed using tools from the ideation and prototyping stage of DT pedagogy. The course ended with final presentations in the thirteenth session. Both groups presented their projects, including the problem they identified and the solution they came up with to solve the problem.

**Fig. 3:**
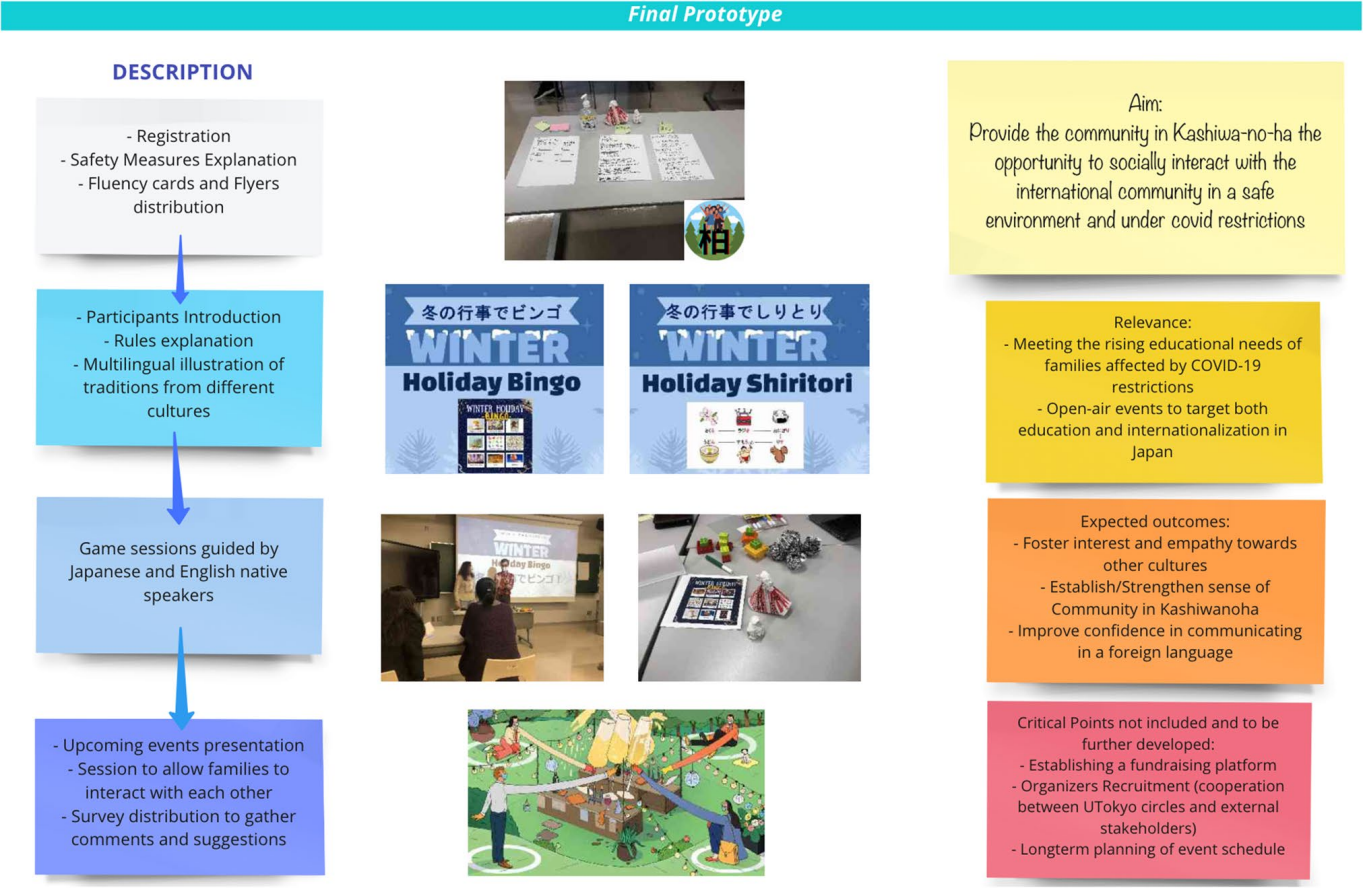
Summary of the prototype on winter holiday event on Miro Board

Out of 13 sessions, three sessions were organized face-to-face and rest of them were online. Two face-to-face sessions were conducted during the empathize stage, where students conducted a field visit of the Kashiwa no ha area and interviewed community members. Students also met experts involved in the development of Kashiwa no ha from the government sector, private sector, and academia. The third face-to-face session was organized during the prototyping stage, where participants brought their ideas into some physical form. For example, during the prototyping session, one of the teams brought their idea of a community engagement office in the physical form by preparing and presenting a role play to demonstrate how will the entity work to engage residents as community members and deal with the problems faced by residents in Kashiwa no ha area. Likewise, the other team brought their idea of a winter holiday event to promote interaction between (local) residents and the international community in the physical form by preparing the props for the event and then running a mockup event. Figure 3 represents the segment of the team’s Miro board with the summary of the prototype for a winter holiday event.

Members of the teaching team, including one faculty member from GPSS-GLI and three teaching assistants from the doctoral program of GPSS-GLI, facilitated the DT pedagogy during FESS. Teaching team ensured that students were involved in the discourse and reflection. Mezirow (1978, 1991) emphasized that educators should support learners to elaborate, transform, and create their meaning schemes through reflection to encourage transformative learning. Therefore, educators took the role of facilitators to support learning. DT pedagogy was flexible and self-directed, where pluralism of thought was encouraged through facilitation instead of concealing it with controlled (transmissive) learning situations. The faculty member was well equipped to facilitate design thinking and was involved in designing the FESS course. Two preparatory sessions were organized to equip teaching assistants with knowledge about DT pedagogy and communicate what is expected of them as course facilitators.

Regular assessments were organized in the form of peer feedback and progress checks. Peer feedback sessions took place at the end of each stage of DT to allow teams to give feedback to each other on the outcomes of each stage. Peer feedback sessions also served as peer assessments. Progress checks were planned to ensure that students were mindful of their position and progress during the course. Each team had to fulfill the progress check requirements of a particular stage and go through the check with the teaching team to proceed to the next stage of DT. Each progress check had different requirements depending on the stage of DT students were in.

Each team was asked to draw their team’s voyage through the DT stages on a journey map, the format for which was provided on their respective Miro boards. The team presented the journey map at each progress check to reflect on their team’s journey during the respective stage of DT.

### Participants

FESS course was open for registration to graduate-level students from different academic departments within the University of Tokyo. Eleven students participated in the FESS course (nine Masters students from GPSS-GLI, one Masters student from Environmental Systems, and one Ph.D. student from Socio-Cultural Environmental Studies). Eleven students were divided into two teams, one team with five members and the other with six members. Teams were formulated based on each student's background (cultural, regional, disciplinary, personality) to ensure that teams were as diverse as possible. The data on students' backgrounds were collected during the course's first session. Then, the teams were formulated based on these data and students worked in teams throughout the course. See Table 2 for more information on the characteristics of the course participants (pseudonyms assigned) and each group's composition.

**Table 2:** Characteristics of course participants (students)

Group	Participant’s Pseudonym	Gender	Age	Academic Background	Regional Background	Department
Group-1	John	Male	24	Natural Science	Asia, China	Environmental Systems
	James	Male	21	Humanities	America, United States of America	GPSS-GLI
	Daisy	Female	22	Natural Science	Asia, Indonesia	GPSS-GLI
	Mary	Female	28	Engineering	Europe, Italy	GPSS-GLI
	Rina	Female	22	Social Science	Asia, Japan	GPSS-GLI
Group-2	Samy	Female	27	Natural Science	America, Brazil	GPSS-GLI
	Arsh	Male	22	Social Science	Asia, Indonesia	GPSS-GLI
	Mina	Female	27	Other—Sustainability Science	Asia, Vietnam	GPSS-GLI
	Jane	Female	23	Social Science	Asia, China	GPSS-GLI
	Naha	Male	27	Others—Natural Science & Social Science	Asia, Philippines	GPSS-GLI
	Yuta	Male	26	Engineering	Asia, Japan	Socio-Cultural Environmental Studies

### Methods and data collection

**Fig. 4:**
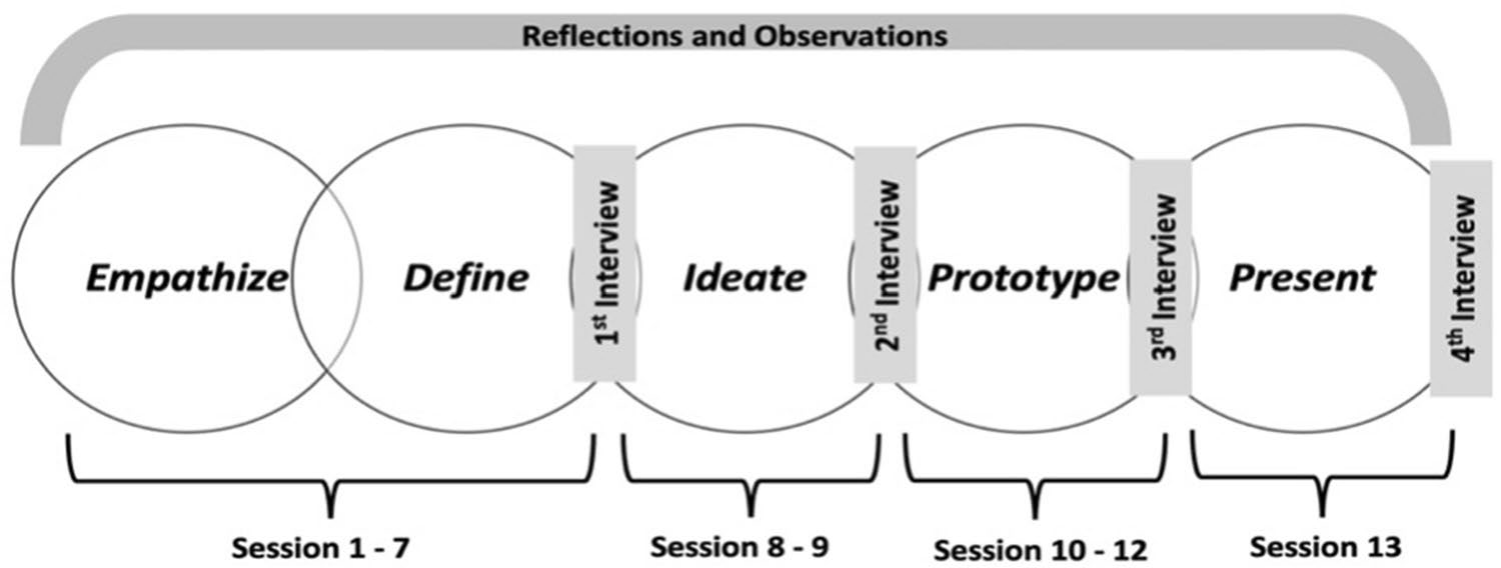
Data collection scheme

The transformative potential of DT pedagogy in the hybrid setting was explored by capturing the signs of transformative learning among the students participating in the FESS course. The signs of transformative learning using THM were captured by exploring the students' perceptions who were attending the FESS course. The purpose of the research guided the selection of research participants to collect qualitative, information-rich data related to the phenomenon under investigation (Palinkas et al., 2015; Suri, 2011). All the eleven students who attended the FESS course participated in the study. Data to explore students' perceptions were collected using semi-structured interviews, open-ended reflections, and class observations (see Fig. 4).

Semi-structured in-depth interviews were conducted four times during FESS (with each participant) through Zoom video call as authors refrained from face-to-face interviews due to the Coronavirus pandemic. Archibald et al. (2019) recommended Zoom as a viable tool for qualitative data collection because of its cost-effectiveness, ease of use, security selections, and data management features. Each interview was 35–45 min long and recorded via the Zoom recording feature. The first round of interviews was conducted after the define stage (after session 7) to acquire students’ perceptions of their experiences during empathize and define stages. The second and third rounds of interviews were conducted after the ideation stage (after session 9) and after the prototyping stage (after session 12), respectively. The last round of interviews was conducted after the final presentations, and it was more focused on reflecting on the overall experiences during FESS. A review conducted by Snyder (2008) and Taylor (2007) also recommended a longitudinal study design to document transformative learning. The starting probes for semi-structured interviews were constructed based on the THM to capture students’ perspectives on their learning experiences while going through the DT pedagogy; probes for the first interview are shown in Table 3.

**Table 3:** Starting probes for semi-structured interview after define stage

Probes for interview after define stage of DT
• Can you elaborate on your overall experience during the Empathize and Define Stages?
o What was it like?
o What did you learn?
o How was the teamwork?
o How did you feel?
o Was there any surprising or unexpected experience?
o Any complexities?
o What did you learn about yourself?
• Do you think your past experiences impacted how to saw experiences/things during empathize and problem framing stages of FESS -Kashiwa-no-ha?—Elaborate
• Have experiences during the empathize and problem framing stage challenged your deeply help assumptions or perspectives?—Elaborate
• Have the experiences during the empathize and problem framing stage allowed you to consider a new perspective?—Elaborate
• Did you adopt any new perspective/s?- Elaborate

FESS participants were also asked to reflect on their experiences after each session during the course and fill out an online reflection sheet. Reflection sheets were created as an internet survey with open-ended questions using Google forms. The research used Mezirow's definition of reflection, also termed as critical reflection, i.e.,

The process of critically assessing the content, process, or premise(s) of our efforts to interpret and give meaning to an experience (Mezirow, 1991; p. 104)

This kind of reflection is mainly associated with adult education, specifically in transformative learning (Mezirow, 1991). Being open-ended can be advantageous to promote critical reflection because, without too many preconceptions on what to focus on, some deeply held assumptions can be uncovered (Fook, 2015). The prompts for open-ended reflection sheets are listed in Table 4. Reflection sheets provided a chance to capture participants' perspectives which could not be captured through semi-structured interviews due to their semi-structured nature and theoretical underpinning. The reflection sheets helped in recording participants' perspectives on their experiences promptly. In addition, they aided the preparation of semi-structured interviews; in some cases, participants were asked to elaborate more on what they had written in the reflection sheet/s during the sessions before the interview. Observations were recorded throughout the course by observing the group discussions and Miro boards in the form of memos and snapshots.

**Table 4:** Prompts for open-ended reflection sheets

Prompts – Open ended reflection sheets
• Name*
• Date*
• How was today’s session? Anything specific you want to share?
• What did you learn today (academically, professionally, or personally)?
• What is/are the key take away/s from today’s session (academically, professionally, or personally)?
• How did you feel today, during the session?

*Compulsory question: only name and date were compulsory to fill in

### Ethical considerations

The current study involved human subjects, and therefore, informed consent was used to ensure ethical research. Informed consent was developed to get an agreement from the participants on their participation in the research, research procedures, research purpose, voluntary nature of participation, measures used to ensure confidentiality, benefits, and risks of research. The research participants had to agree to the informed consent to participate in interviews and access the reflection sheets. Data were collected, anonymized, and stored in a password-protected system, accessible to the authors only.

### Data analysis

The signs of transformative learning among the students were investigated based on THM through thematic analysis of the interview, reflection data, and observation notes (Boyatzis, 1998; Braun & Clarke, 2012) using the MAXQDA software. Thematic analysis encodes qualitative data by systematically identifying, organizing, and offering insight into the pattern of themes across the data set (Braun & Clarke, 2012). Initial coding was deductive using research-driven categories based on THM, as visually presented in Fig. 2. The second round of coding was inductive. See Table 5 for an elaborated explanation of the data analysis steps using thematic analysis (Boyatzis, 1998; Braun & Clarke, 2012; Kuckartz­ & Rädiker, 2019; Pearse, 2019).

**Table 5:** Steps performed to analyze the data, using thematic analysis

S.no.	Data analysis step	Explanation
1	Transcription and labeling	All the recorded data, from semi-structured interviews, reflection sheets, were transcribed and transcripts were generated. Data in the form of transcripts, observation memos, and pictures (snapshots) were labeled
2	Familiarization	All transcripts, memos, and pictures were examined twice to immerse in the data, and then, they were examined again for further immersion and for preparing memos
3	Codebook	A preliminary codebook was developed in MAXQDA with codes based on THM. Each code’s label, definition, and description were recorded. 7 codes were determined based on THM
4	Initial coding	The codebook is applied to the collected data. This involves reviewing, revising, and confirming that the codes do appear in the data by collecting examples
5	Adding codes	Data were analyzed again to explore additional codes (inductive coding) to extend the analysis beyond the theoretical propositions derived from THM. 15 additional codes were added to the codebook
6	Identifying Themes	Themes were identified by exploring patterns in the data to capture the signs of transformative learning based on THM by connecting codes to one another. Initially, 9 themes were identified
7	Reviewing	Themes identified were reviewed in relation to the coded data and entire data set to verify that the themes finalized were aligned to and compatible with the data set. After reviewing, 9 themes were collapsed into 6 themes

The study used Intercoder agreement, where independent coders evaluated the characteristics of a message, and the same conclusion was reached (Silverman, 2005; Tinsley and Weiss, 2000). The first author analyzed the data, followed by an independent data analysis by the third author, and then, the second author checked the themes aligned to the codes. There were no significant differences, and the minor discrepancies were discussed and resolved to create the set of themes presented in the paper. The finalized themes and aligned codes are represented in Table 6.

**Table 6:** Finalized themes and aligning codes and the number of coded segments

Theme	Code	Coded Segments
Transformation Helixes	Experience based assumptions	177
	New experiences	95
	Challenging perspectives	112
	Transformations	235
Reflection Capacity	Reflections	36
	Sharing experiences	72
	Understanding perspectives	61
	Benefits of critique/feedback	10
State of Comfort	Hard	91
	Uncertainty	16
	Comfortability	11
	Evolution of teamwork	36
	Teamwork and efficiency	45
Engagement	Fun	42
	Satisfying	61
	Drive	7
Sense of Attachment	Attachment	15
Course Organization	Organization	15
	Visualization	4

## Results

This section presents the six themes prevalent in the data, probing participants’ perspectives on their experiences during the FESS course to explore the transformative potential of DT pedagogy in the hybrid setting. The themes which emerged from the data include (a) The Transformation Helixes; (b) Reflection Capacity; (c) State of Comfort; (d) Engagement; (e) Sense of Attachment; and (f) Course Organization. These themes are explained as follows:

### The transformation helixes

The data analysis captured signs which demonstrated that participants experienced transformative learning phases aligned to THM throughout the implementation of DT pedagogy. These learning phases were experienced in a sequence, i.e., experience-based assumptions were followed by individuals challenging their own perspectives (when encountered with new experiences provided by DT pedagogy) and changing their perspectives or adopting new perspectives. The signs of experiencing all these learning phases were captured in all stages of DT.

Participants' previous experiences affected the way they perceived learning experiences, initially, during all the stages of DT pedagogy. These past experiences mentioned by the participants were not only personal but also professional/academic experiences. James:

I would say the biggest thing was in the empathize phase when we had all these people we were talking to and all my conceptions of what it would be like[…] I expected them to talk about changes that I have experienced during Coronavirus, and they just gave completely different answers[…]okay, all my conceptions are just wrong, I kind of realized that….

While going through the DT pedagogy, participants became aware of their biases. Further, they understood that these biases play an essential role in defining their worldview—that they viewed their learning experiences from the lens of their past experiences (initially), during each stage of DT:

I had this idea…oh smart city is just a gentrification. I went there with an idea, very critical about the corporations or the private sector, I went with that bias… (Naha)

DT pedagogy provided new (learning) experiences that allowed participants to challenge or question their initial assumptions based on their past experiences. Most of these new (learning) experiences involved discourse where participants communicated, e.g., group discussions, interviews with the stakeholders, team coordination, and presentations. Daisy:

I saw the problem in more economic and financial issues but after the group discussion, they also mentioned about the energy preservation and also about education[…]Yeah, it also changed my perspective.

According to the observations conducted during the classes, the new experiences involving discourse encouraged the participants to step out of their comfort zones. Participants struggled to either explain their perspectives or understand others' perspectives. As a result of these new experiences, the course participants mentioned that their perspectives changed. Arsh, during the last interview, said that based on his past experiences, he believed that Japanese people do not care about community engagement but after going through the course and engaging with Kashiwa no ha community, his perspective on the Japanese community changed, and he stated:

I think generalizing a community in a certain way is not correct. I think this experience really changed my perspective…

The participants simultaneously went through multiple transformative learning experiences (transformative helixes), cognitive and affective. Therefore, the transformative learning experiences provided by DT were multidimensional. For example, one of the research participants (John) stated:

I have never been involved in the community before, actually listening to the Matsumoto san's (stakeholder's) family, it made me really want to get involved in the community aspect. So I have been trying to talk to people around the routes, which I usually kind of get silent. So, it made me a lot more open to others in my daily life...

The same participant, during the same interview, also quoted:

I have never seen myself much as a leader. Then in the problem framing phase, when it was really down to teamwork and group cohesion, I had to take the lead […] I think during this process I really learned how to lead…

By the end of the course, some participants acknowledged that the changes they had encountered in them are their new reality or experiences. James:

Some of my own past experiences now doesn't make sense, where I used to be. Just because I gained more experience through the course.

**Fig. 5:**
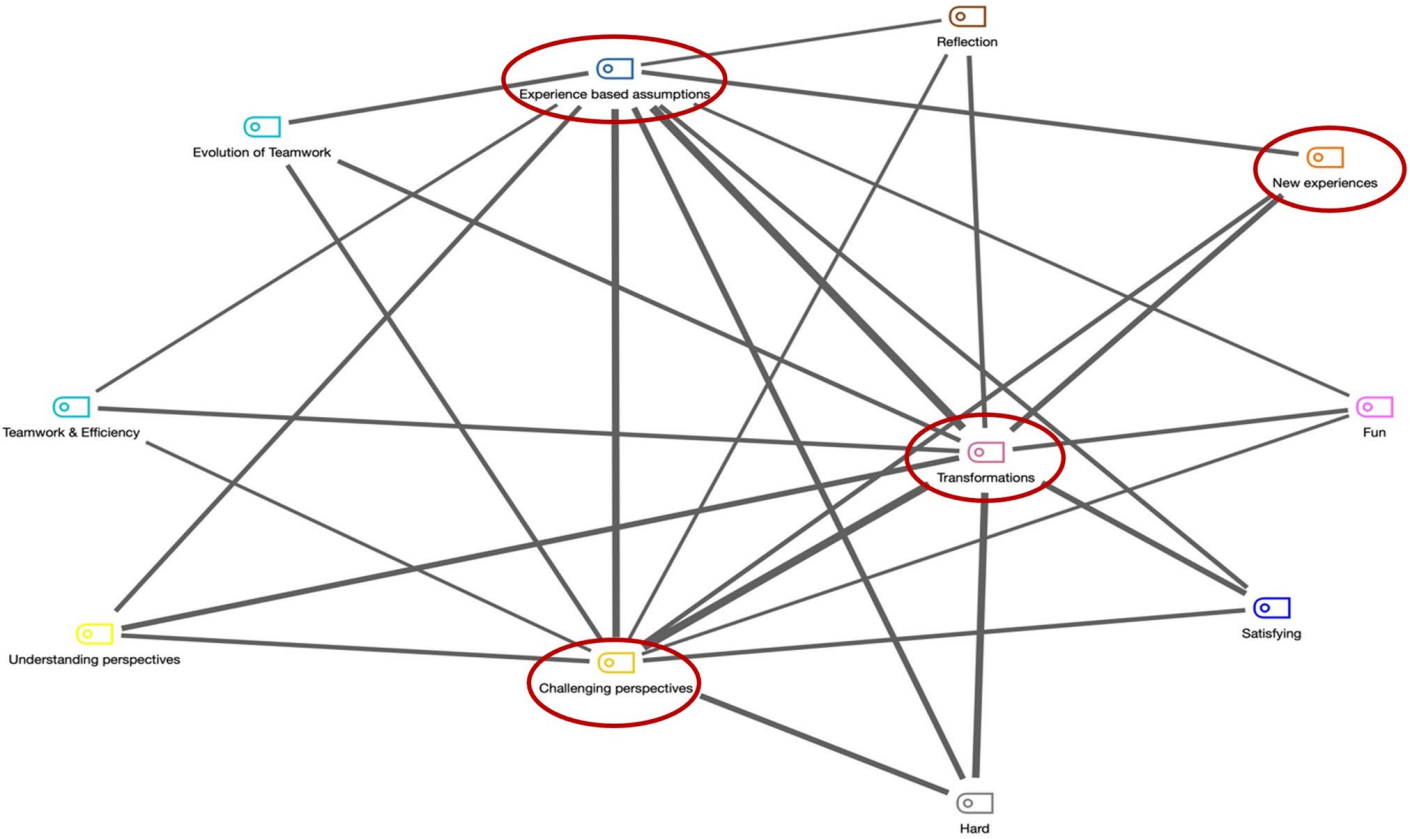
Code co-occurrence model extracted from MAXQDA

Participants' perceptions data highlighted multiple signs indicating that they had experienced learning phases demonstrated by THM. The code co-occurrence model extracted from MAXQDA (Fig. 5) also explains the co-occurrence of codes aligned to THM. The code co-occurrence model demonstrates how many documents have co-occurring codes aligned to THM. While building this model, the minimum co-occurrences were set at 14 (codes co-occurring in more than 14 documents were displayed). The width of each linking line represents the frequency of co-occurrence of codes in the data (documents). The width of the connecting lines in the figure reveals that the transformation code is frequently co-occurring with experience-based assumptions, challenging perspectives, and new experiences. The code co-occurrence model shows that the codes aligned to THM occur together. However, it does not highlight that they were occurring in sequence, which was apprehended by the quotes from the participants (as explained above).

### Reflection capacity

As participants progressed through different stages of DT, their reflection capacity kept enhancing as they were frequently reflecting (practicing). In the first two stages, the responses to the interview questions and reflection sheets were not as reflective. However, as they progressed into the following stages, they started reflecting in detail and tried analyzing the situation in depth. Participants also saw the benefits of reflection, as Naha stated:

[...]because I never do reflection about myself that much but that's why I like this exercise that we do self-reflection, because it makes me more mindful of my own way of thinking, so that is probably one of the reasons why I'm now more aware of myself.

The enhanced capacity to reflect allowed participants to think about and explain the reasons for choosing particular perspectives, behaviors, or actions (making sense-professionally/academically/personally), which enhanced self-awareness among the participants. Samy explained why she did not want to build her career in academia, which she found difficult to reason and explain earlier:

[...]it was always hard to elaborate[…] during the course, I realized that maybe because in our work as researchers, it's pretty much one person job. I feel it doesn't give me 100% joy. So, I feel that I work better if I am in an environment where you can talk to people and really do it together, and also identify a problem and do something about it that is probably going to be implemented.

Reflection capacity has played a significant role in making participants acknowledge that they have certain perspectives that originate from their past experiences (self-awareness) and that their fellow teammates have their own perspectives. In addition, the recognition of positions kept enhancing among the participants as they progressed through the course. Hence, in the empathize and define stages, they understood that other people in their team have different perspectives compared to their own perspectives. Yuta highlighted that he realized that everyone in his team comes from different background, and that is why everyone has a different view and said:

[…]so when someone speaks up during the discussion the other will add[…] like I will say in my country, and Samy will say in Brazil, Mina will say in Vietnam, and that's very interesting.

In the ideate stage, participants started to recognize that their perspectives might differ from the perspectives of their teammates, and it is difficult to not have conflicting ideas in the team. Rina while talking about one of the conflicting discussions said*:*

Firstly, I felt kind of nervous, but at the same time, I thought that it's a natural thing, because everyone has different academic background or way of thinking.

The acknowledgment of their own and their teammates' positions regarding perspectives made participants recognize the value of multiple perspectives and be more open to new perspectives and ideas. Mina mentioned:

I like implementing stuff or doing things rather than having a theoretical background or having a nice framework. But when talking to other people in the class, I can see how we learn from each others' strengths. Some are good at theoretical background like abstract thinking, and someone is better at doing things. So, somehow we can complement each other.

### State of comfort

Participants felt uncomfortable, overwhelmed, confused, surprised, and even in self-doubt during the initial stages of DT, precisely the define stage. However, the state of comfort kept improving as they progressed further to the ideate and prototyping stages. Participants found the define stage (problem framing), one of the earlier stages, to be the most challenging and most uncomfortable stage of DT pedagogy because pinpointing the sustainability problem required participants to narrow down the scope of the problem, which exposed them to uncomfortable confrontations. In addition to uncomfortable confrontations, some participants mentioned that the initial stages were difficult because they had just started teamwork and did not understand their team members well. Jane:

The most difficult stage I think is the problem framing because at this stage we have so many problems, each of us we have different opinion and sometimes the confrontation process is long and painful.

**Fig. 6:**
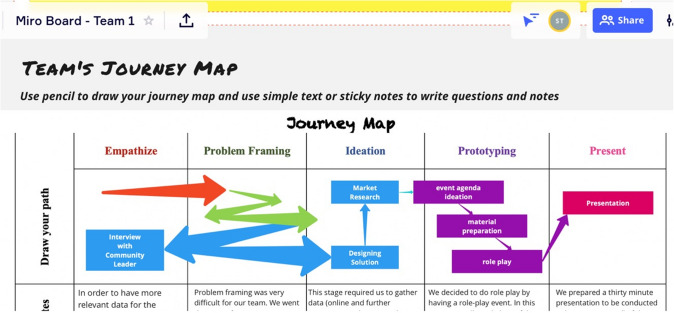
Team-1’s journey map showing their progress through DT stages

The difficulty of the problem framing phase is also evident from one of the teams' journey map (see Fig. 6) which demonstrates the team's progress through the DT pedagogy. The journey map indicates a lot of back-and-forth movement, specifically during the problem framing stage.

During the ideate stage of DT, the participants felt more comfortable while going through the stage than the empathize and define stages. Even though one of the teams had to revise the framing of the problem and had conflicting ideas when working together, they still felt that the ideate stage was less complicated. Arsh emphasized:

So, ideation phase compared to the first two phases, is not that confusing or stressful. And yeah of course, there are many ideas in our group and a lot of clashing ideas. But I mean, as usual, we always could come up with a consensus.

Participants' familiarity with DT and the team members (enhanced team bonding) made going through the DT pedagogy comfortable. Data showed that team members took some time to understand each other. When they started teamwork, there were times when they felt discomfort while working with each other. For example, Jane said it was difficult to understand her teammates initially; therefore, she would take things personally. However, (gradually) as the team bonded well, that was no longer an issue. Teamwork kept evolving as participants progressed through the stages of DT and team members started feeling comfortable with each other. Even quieter members of both teams started voicing their opinions which further contributed to team bonding. The cohesion of teams enhanced teams' efficiency and engagement in the course. A research participant (Naha) cited in the reflections*:*

I was also extremely happy as well that the group dynamics have improved, the used-to-be quiet ones are more vocal and active this time. I think the team is now becoming more and more aware or sensitive about other members, which made our group dynamics today even more productive and engaging.

Figure 5 also highlights that team evolution, and team efficiency codes co-occur with the codes aligned to THM, i.e., experience-based assumptions, challenging perspectives, and transformations. This underpins the importance of team building leading to team efficiency to ensure that participants can maximize their transformative learning experience.

During the FESS course, the participants worked in the same team from the beginning till the end, and that allowed them to collaborate radically across differences as they progressed through the course. Working in the same team throughout the course led to forming friendships and attachment among the team members, and it was evident specifically in the last interview:

I not only got the credits for the course but also got many friends, and I don't think this is common in many other courses. (Jane).

### Engagement

Apart from finding the learning experience challenging, the participants also enjoyed the experience. The feeling of satisfaction, fun, and enrichment while experiencing DT enhanced participants' engagement and motivated them to look forward to the following stages of DT.

In the first two stages of DT pedagogy, most participants realized that the learning experience was fun after going through the empathize and define stages, not while experiencing these stages. A participant stated:

[...]it was challenging for me, at the same time exciting because it was my first time to actually sort of conduct an output[...]Nice experience for me! (Naha).

When participants moved on to the following stages, they started appreciating the learning processes and the outcomes. Participants started enjoying the brainstorming and discussion sessions and expressed that in their reflection sheets:

The discussion was fun and interesting. Everyone was engaging and active in the discussion. Many new ideas have been initiated from our group work today[…] (Mina)

Participants also felt satisfied after seeing the outcome of each stage of DT, which enhanced their motivation to stay engaged. Mary wrote in the reflection sheet:

I am happy with the final result, so I'm now satisfied.…. I also hope we will manage to proceed smoothly to the next stage!

### Sense of attachment

After framing sustainability challenges in the Kashiwa no ha area and ideating solution ideas to deal with these challenges, participants expressed a sense of attachment to the community. Participants showed genuine intention to solve the sustainability challenge their group had framed. This attachment initiated a sense of responsibility and a sense of belonging to the community. James said that their team got attached to the community after proposing the holiday events solution, and they wanted to do these events to address the need for community building and internationalization:

[…]I really feel bad about sending out this information to people getting their feedback, and not carrying out like an actual thing for this holiday and we were like, should we form a circle and do this? […] in doing this project, we have gotten attached to the community. It felt more like we belong here. (James).

After the prototyping stage, another participant said:

I'm very happy if our solution idea can be implemented somehow. We could try to solve the social issues in the area by our idea. (Arsh)

### Course organization

Participants gave feedback on the organization of the course and to improve it further. The FESS course was organized using a hybrid learning approach for the first time, with most of the work organized in the digital environment. Participants called it a success and appreciated how it was organized digitally. Samy:

In the beginning, I thought, how can we do this on zoom, right, because I think when I think about group work, I feel the image that comes to my mind is, let's say, a white paper and post its and conversations. So, I thought it would be very hard. However, now I feel that this has worked. I was literally thinking it's not going to work online. So, I was surprised that we could, because 90% was online work.

Participants found the course demanding and challenging, and most of the participants mentioned that during the last interview. Participants suggested making it either a two-semester-long course or increasing the credits for the course:

I like to emphasize how demanding this class was it was it was not only demanding of like time but emotionally as well. (James).

Participants also appraised the Miro board as a tool to work in groups as James said:

Miro board, I've never even heard of that. Moreover, that was probably something you continue to carry on, just because it's so good for helping teams function.

Through Miro boards, participants saw the value of visualizing information and thinking process. However, in an online learning environment, Miro boards made the visualization possible. Visualization enabled the participants to engage in discourse constructively, reflect effectively, and see their progress till the end. Rina:

I learned it is meaningful to try to visualize the thinking process and reflect on our work from the beginning to the final part because it will be easy to go back to previous stage (design thinking in this case) when we have some problem to do the work.

## Discussion

The purpose of the current research was to explore the transformative nature of DT pedagogy using THM, in HSE, by capturing the signs of transformative learning experiences. The results have demonstrated that participants have experienced the THM's learning phases during each stage of DT pedagogy. Furthermore, the detailed experiences of the participants apprehended by interviews, reflection sheets, and observations revealed that learning phases were sequential. Participants mentioned that their perspectives were built on their past experiences (personal/professional), and then, these perspectives were challenged after going through the experiences provided by DT pedagogy.

According to Mezirow (1990, 1991), perspectives are composed of beliefs, values, and assumptions—acquired through experiences, which define our worldview. Although these perspectives are helpful, sometimes they can be biased and flawed, distorting our ability to be open to new perspectives and ideas. Mezirow added that we could identify, evaluate, and transform (modify) our perspectives through discourse and critical reflection. Where discourse and critical reflection are supported or triggered by stimulating experiences, also called disorienting dilemmas (Mezirow, 1991). The results indicated that DT as a pedagogy provided disorienting dilemmas (in the form of new learning experiences), which brought in the elements of critical discourse where participants reflected upon the subject and situation with others and questioned their own perspectives. Disorienting dilemmas are the experiences which drive individuals to question their own perspectives and go through perspective transformation (Mezirow, 1978, 1991). Disorientation provided by DT pedagogy encouraged learners to experience perspective transformation as the signs of changes in participants' perspectives appeared in the results. These changes in perspectives were either revisions in their previous perspectives or acquiring a completely different perspectives. The code co-occurrence model extracted from MAXQDA (Fig. 7) confirmed that the codes allocated to learning phases demonstrated by THM significantly occurred in most documents (interview transcripts, observation notes, and reflection sheets). The participants regarded the changes in their perspectives as their new reality (or new experiences), indicating that once transformative learning occurs, it is unlikely that adults revert to their prior beliefs (Nerstrom, 2014). Therefore, transformations are unidirectional, and they are demonstrated as helixes in THM and not as a cycle (circle).

It was observed that participants went through multiple helixes of transformations, both cognitive and affective (multidimensional), at the same time. A study conducted by Damianakis et al. (2019) also revealed that the transformative learning process and outcomes were multidimensional and personified in origin. All the participants of the FESS course went through the learning phases aligned to THM, but experiences and changes in perspectives were different for every participant. Oxenswärdh and Persson-Fischier (2020), while implementing DT in HSE, also observed that students learned together (in the same group) but did not learn the same thing and considered that as collaborative learning.

Results indicated that the DT pedagogy made participants more reflective. This reflection capacity kept enhancing as they progressed through the stages of DT. The capacity to reflect allowed the participants to make sense (be self-aware) of their own perspectives and the perspectives of their teammates. Neergaard et al. (2020), while investigating the role of pedagogical nudging and reflection, also reported heightened self-awareness among the participants and recognition of the importance of others. When participants can give perspective about their own perspectives, this sets up the condition for transformative learning (Mezirow, 2003). Hence, reflection is the critical component of transformative learning (King, 2018; Taylor, 2007). Macintyre et al. (2020) also highlighted diversity (multiple perspectives) and reflexivity (about who we are) as the foundation for transformative learning. Furthermore, the study found that acknowledging multiple perspectives and their value made the participants more reflective, inclusive, and open to new/different perspectives—aligned to the definition of transformative learning proposed by Mezirow (2003).

Having an open mind, listening empathetically, avoiding judgments, and seeking common ground are necessary conditions to participate in critical discourse for transformative learning (Mezirow, 2003). The participants' openness to new/different perspectives encouraged engagement in critical discourse. Consequently, the learning experiences kept becoming comfortable for the participants as they progressed through the stages of DT pedagogy. The initial stages of the DT, i.e., empathize and problem framing, were tough. Participants felt frustrated and confused because of the iterative nature of the initial stages, a new way of approaching the problem, and students requiring time to become a team (Oxenswärdh & Persson-Fischier, 2020). As participants progressed to the ideation stage, they started getting comfortable with the DT pedagogy and their team members. Participants established trusted relationships with their team members, which created a comfortable space for appreciation and effective engagement in critical discourse. To ensure that participants feel comfortable sharing their beliefs, assumptions, and vulnerabilities, being in a state of comfort is crucial. Comfort is a prerequisite for experiencing discomfort, ambiguity, and transformation (Wolgemuth & Richard Donohue, 2006). Taylor (1998, 2007) also mentioned establishing trustful relationships to share openly, one of the essential factors for a transformative experience.

The study found that participants kept challenging their own assumptions throughout the course multiple times. Challenging perspectives is not a comfortable learning phase (Macintyre et al., 2020; Roberts, 2006); therefore, it evoked various emotions among the participants. Hence, they found the course enjoyable but challenging at the same time. While being challenging, DT engaged the participants effectively, and these engaging experiences were reported as fun, satisfaction, enjoyment, and enrichment. Engagement in the course ensured significant exposure to DT as a transformative pedagogy. Moreover, enhanced engagement, collaboration (teamwork), and active learning (problem-solving) can foster transformative learning (Hassi & Laursen, 2015).

Through DT, participants got an opportunity to deal with real-life sustainability challenges and developed responses to these challenges in the Kashiwa no ha community. Kashiwa no ha community was selected as a case to set the context. The capacity to learn in context (place-based learning) adds layers of meaning and understanding to transformative sustainability learning by bringing together the sustainability content, experiential learning, and multiple perspectives, all rooted in a specific geographical place (Burns, 2011). Taylor (2007) highlighted the role of context in shaping the transformative experience. Although none of the participants was from Kashiwa no ha community, through DT pedagogy, participants can relate to the community and felt belonged to the community by reflecting on their positions in the community. Participants showed the signs of deep reflection involving connecting to the community and reflecting on their purpose or responsibility toward the community. As a result of this context setting, participants expressed the desire to do something about the challenges in Kashiwa no ha and stay engaged with the community to implement their proposed solutions even after the FESS course finishes. Transformative learning ensures capability and motivation among learners to contribute to the social good (King, 2018). In HSE, transformative learning is manifested as intentions to make a difference in their communities, promoting sustainable actions (Piasentin & Roberts, 2017; Probst et al., 2019).

Although the FESS course was conducted using a hybrid learning approach, visualization of information, ideas, and thinking processes was guaranteed through Miro boards. One of the critical principles of DT pedagogy is visualization, which proposes to make abstract ideas visible and tangible (Buhl et al., 2019). Visualization endorsed reflection, effective engagement in discourse, and appreciation of project outcomes among participants.

## Implications

This section lists down the pedagogical, theoretical, and empirical implications of the research based on the reflection done by researchers on the research findings.

As a pedagogy, DT enables learners to go through transformative learning experiences. For education practice, the current study proposes that DT may bring transformative learning into practice in HSE in hybrid settings. Amid the COVID-19 pandemic, many higher education institutions shifted from face-to-face teaching to online synchronous and asynchronous teaching (Carolan et al., 2020)—making higher education more transmissive. According to Krishnamurthy (2020), the impact of the pandemic will bring an era of technological transformation and accelerate the digitalization of higher education across the globe. Therefore, universities need to re-think and re-design their pedagogical approaches to encourage transformative learning while facing the new era of technological transformation. To prevent the spread of the COVID-19, DT pedagogy was implemented in a hybrid format during the FESS course, and most of the sessions were organized online. DT pedagogy, in hybrid settings, has successfully worked in bringing transformative learning into practice without complex structural changes. This study provides an example of an effective, transformative pedagogy that can be used in HSE to ensure human development suited for sustainable development, even in digital settings.

The transformative learning experiences of the participants going through the DT pedagogy were personified and embodied. Without individual transformation, social change for sustainability is impossible because if the personal transformation has genuinely taken place, it is impossible to prevent its impact on the social context (Mezirow, 1995; Servage, 2008). Therefore, if the objective is to educate leaders who can deal with sustainability challenges, HSE shall use the proper pedagogy to achieve the transformative goal rather than simply imparting knowledge (or building anxiety) about sustainability challenges.

Working in diverse teams is vital for implementing DT pedagogy, as Munyai (2016) and Rauth et al. (2010) elaborated. Therefore, the FESS course was open to (graduate level) participants from all departments. However, the course was organized in English to ensure that learners, irrespective of their background, can effectively engage in discourse. Setting up the course in the English language helped participants from different backgrounds to engage in radical collaboration and critical discourse. However, the organization of the course in the English language in a Japanese university automatically filtered out participants who were not fluent in the language. This highlights the need for future research to explore how diversity in teams can be ensured when the course using DT pedagogy is not organized in English.

The study explored transformative learning as a process while implementing DT as a pedagogy, which can inspire future educational programs in the digital setting. The THM acts as a means to analyze data and monitor learning processes. Theoretically, the basis to investigate the transformative nature of DT pedagogy was THM based on Nerstrom's (2014) learning phases. THM visualized the transformative learning process, and the current study captured the signs of transformative learning aligned to THM learning phases. However, in reality, transformative learning is much messier than what the model represents. Therefore, it would be helpful for future research to investigate the transformative nature of DT pedagogy in different contexts in HSE using THM.

Empirically, the research used the longitudinal study design to capture signs of transformative learning where data were collected multiple times, using multiple tools, during the implementation of DT pedagogy. Data were collected from the same participants over time, which helped researchers understand the process of transformative learning and how signs of transformative learning evolved with time. The challenge of the longitudinal study is separating between what is related to transformative learning experiences and what is the product of normal development of individuals or/and changes in the society (Taylor, 2007). To overcome this challenge, the semi-structured interviews included questions specific to the course components and participants' learning experiences. While the semi-structured interviews included disruptive questions (questioning what participants have experienced), they also integrated empathetic questions (to understand participants' experiences). Furthermore, an open-ended (emancipatory) approach was taken to collect data via reflection sheets. In this way, the researchers took a balanced approach (between disruptive and empathetic), which aligns with Macintyre's (2020) recommendation to explore transformative learning as a process. Future research exploring transformative learning as a process would be wise to note that more balanced methodological approaches are needed to gather in-depth and rich data from the participants. To ensure a balanced approach to data analysis, researchers used deductive and inductive approaches to analyze the data—to capture the signs of transformative learning aligned to THM. However, they did not restrict the identification of signs to the theoretical model (only).

## Conclusion

This paper suggests that DT as a pedagogy sets up the learning environment and processes to promote transformative learning experiences in HSE. The study successfully captured the signs of transformative learning (via participants’ perspectives) aligned to THM during the FESS course at the University of Tokyo, using DT as a pedagogy. The signs revealed that the learning phases, i.e., experience-based assumptions, challenging perspectives, and perspective transformations aligned to THM, were experienced in a sequence. Each participant went through embodied and multiple helixes of the transformative learning experience simultaneously. Other signs of transformative learning that appeared in the data analysis showed that DT as a transformative pedagogy made participants more reflective, collaborative, engaged, responsible, and attached to the community. Hence, the study reveals that DT pedagogy can support transformative learning in HSE by providing disorienting dilemmas, encouraging reflection and discourse, fostering relationships, providing context, and offering an engaging experience.

## Footnotes

1 Transmissive approach is defined as the transmission of knowledge by the educator to the learners while disregarding learners’ lived experiences and contexts (Bradshaw, 2017).

2 Learning processes and learning environments are referred to as pedagogy (Siraj-Blatchford et al., 2002; Taimur, 2020, 2022).

3 Diverse teams are the teams with team members from different regional, disciplinary, and personal (gender/age/personality) backgrounds.
